# A Portable Molecularly Imprinted Sensor for On-Site and Wireless Environmental Bisphenol A Monitoring

**DOI:** 10.3389/fchem.2022.833899

**Published:** 2022-02-16

**Authors:** Tutku Beduk, Matilde Gomes, José Ilton De Oliveira Filho, Saptami Suresh Shetty, Walaa Khushaim, Ricardo Garcia-Ramirez, Ceren Durmus, Abdellatif Ait Lahcen, Khaled Nabil Salama

**Affiliations:** Sensors Lab, Advanced Membranes and Porous Materials Center (AMPM), Computer, Electrical and Mathematical Science and Engineering (CEMSE) Division, King Abdullah University of Science and Technology (KAUST), Thuwal, Saudi Arabia

**Keywords:** laser-scribed graphene, electrochemical sensors, molecularly imprinted polymers, bisphenol A, environmental monitoring, KAUSTat

## Abstract

The detection of pollutant traces in the public and environmental waters is essential for safety of the population. Bisphenol A (BPA) is a toxic chemical widely used for the production of food storage containers by plastic industries to increase the storage ability. However, the insertion of BPA in water medium leads to serious health risks. Therefore, the development of low-cost, practical, sensitive, and selective devices to monitor BPA levels on-site in the environment is highly needed. Herein, for the first time, we present a homemade portable potentiostat device integrated to a laser-scribed graphene (LSG) sensor for BPA detection as a practical environmental pollutant monitoring tool. Recently, there has been an increasing need regarding the development of graphene-based electrochemical transducers (e.g., electrodes) to obtain efficient biosensing platforms. LSG platform is combined with molecularly imprinted polymer (MIP) matrix. LSG electrodes were modified with gold nanostructures and PEDOT polymer electrodeposition to create a specific MIP biomimetic receptor for ultrasensitive BPA detection. The sensing device has a Bluetooth connection, wirelessly connected to a smartphone providing high sensitivity and sensitivity (LOD: 3.97 nM in a linear range of .01–10 µM) toward BPA. Two commercial bottled water samples, tap water, commercial milk, and baby formula samples have been used to validate the reliability of the portable sensor device.

## 1 Introduction

Bisphenol A (BPA) is a well-known chemical and main ingredient for various plastic products in industries, such as epoxy resins, polycarbonate (PC), and PVC plastics, as containers of both food and beverages (Safe, 2000; Im et al., 2016; Santonicola et al., 2019). BPA may also be found in water pipes, dental sealants, food packaging, dyes, and tanning agents (Chen et al., 2016). The chemical structure of BPA deeply resembles and mimics the estradiol structure, a crucial hormone for the reproductive system (Cobellis et al., 2009; Latif et al., 2014; Arul et al., 2021). This leads to a side reaction that keeps BPA in the body as a production of hormones. BPA has been found to decrease the secretion of estradiol and testosterone (Chen et al., 2016). Exposure to BPA has been proven toxic in various age groups worldwide causing crucial health concerns, such as prostate and breast cancer, development disabilities, and heart disease (Mielke and Gundert-Remy, 2009; Pupo et al., 2012). As well as the health effects, BPA has also known as a threat to the environment. The contamination of the water sources, marine, and underground waters, through the propagation of wastewater treatment plants, landfill sites, and industrial effluents have become a huge concern over the past decade. Since most of the environmental pollution consists of plastics and microplastics, BPA is found in freshwater samples (Huang et al., 2012; Campanale et al., 2020). Studies have found that BPA concentrations are higher in highly developed industrial and commercial regions, compared with relatively rural areas (Huang et al., 2012). BPA has also been detected in sediment and sewage sludge. Due to combustion processes, a certain amount of BPA in the air has also been observed (Huang et al., 2012; Vasiljevic and Harner, 2021). Thus, the development of accurate and reliable sensing system for the detection of BPA in industrial fields has great importance in human health and the preservation of the environment.

Different detecting techniques, including electrochemical and analytical methods, have been previously used for BPA detection (Grumetto et al., 2008; Miao et al., 2014; Ghanam et al., 2017; Ben Messaoud et al., 2018; García-Córcoles et al., 2018; Yin et al., 2018; Lee et al., 2019). However, onsite monitoring holds a great importance for environmental sampling (Ali et al., 2020). Electrochemical detection methods provide practicality as well as accuracy and cost effectiveness. In the last decade, the sensor development based on electrochemical sensing strategies has received a high demand due to the high possibility of having flexibility and mobility. Carbonaceous nanostructured materials have provided high compatibility with various chemicals, and these materials have been excessively used for sensor fabrication (Govindasamy et al., 2018; Nehru et al., 2021; Rajaji et al., 2021). Graphene is known for its high mechanical stability, surface area, electrical and thermal conductivity, and mechanical flexibility. Laser-scribed graphene (LSG) electrodes can be produced by scribing on a polyimide sheet with CO_2_ laser (Lin et al., 2014). LSG electrodes have been previously used for gas sensors, detection of biomolecules, proteins, biomarkers, and neurotransmitters (Fenzl et al., 2017; Ghanam et al., 2020; Lahcen et al., 2020; Beduk et al., 2021a). Electrochemical sensing of BPA requires the use of a working electrode, which is modified by different nanomaterials such as polymers, metal nanoparticles, and/or their combinations (Alam and Deen, 2020; Bas et al., 2021). Molecularly imprinted polymers (MIPs) are synthetic polymers produced via the polymerization of monomers and crosslinkers for the detection of a specific target molecule (Belbruno, 2019; Lahcen and Amine, 2019; Karthika et al., 2021; Xu et al., 2021; Zhang et al., 2021). The combination of molecularly imprinted polymers (MIPs) with LSG electrodes could provide a suitable functionalization platform for sensing applications (Beduk et al., 2020; Marques et al., 2020). MIP-based electrochemical sensors show robustness, high stability, sensitivity, and selectivity due to the specific template fabrication toward the target analyte, providing easy recognition and reusability of the sensing platform (Lahcen et al., 2017). Moreover, the presence of a nanoparticle layer, such as gold nanoparticle (AuNP) modification prior to MIP electrosynthesis, provides a stable electroactive support for EDOT (3,4-ethylenedioxythiophene) polymerization in the presence of a template molecule (Lahcen et al., 2021).

LSGs have been previously demonstrated for BPA detection (Beduk et al., 2020). However, previous reported sensors depend on the use of bulky potentiostats, which is not applicable and practical for on-site applications. Herein, for the first time, all electrochemical measurements were conducted by a portable, custom-made, and wireless potentistat device for on-site environmental monitoring, compared with previous BPA sensors coupled with bulky electrochemical systems. The device consists of a homemade portable potentiostat connected wirelessly to a smartphone and a flexible LSG-MIP-based electrochemical sensor. In addition, compared with the previously reported bare polymer matrix on the LSG surface (Beduk et al., 2020), having a gold nanoparticle layer supported by a polymeric matrix serves a highly electroactive role for BPA detection. The practicality of the device allows users to perform on-site detection of BPA in environmental samples with no requirement for sample pretreatment, while the gold combined polymeric matrix provides ultrasensitive BPA detection. The high-quality LSG sensor combined with MIP matrix in gold nanoparticle–polymer matrix provides higher selectivity and sensitivity with an LOD of 3.97 nM, compared with the commercial potentiostat device (LOD:15 nM). The sensor surface characteristics, such as morphology, chemical composition, etc., were investigated using physiochemical characterization techniques. The electrochemical performance of the developed sensing device is compared with the commercial potentiostat. Detection performance of the sensing device has been validated by measuring BPA in commercial water, tap water, milk and baby formula samples, as well as the commercial plastic samples with successful recovery rates.

## 2 Experimental

### 2.1 Materials and Apparatus

Polyimide (PI) material with a width size of 12” was purchased from Utech Products, USA. For buffer preparation, hexaammineruthenium (III) chloride {[Ru(NH_3_)_6_]Cl_3_; 98%}, potassium chloride (KCl), potassium ferrocyanide K_4_[Fe(CN)_6_], and potassium ferricyanide K_3_[Fe(CN)_6_] were purchased from MP Biomedicals. Phosphate-buffered saline (PBS) tablets were purchased from Fisher Bioreagents. For the solvent preparation, dimethyl sulfoxide [(CH_3_)_2_SO] was purchased from SupraSolv, Merc. Methanol was obtained from VWR company (certified ACS; 99.9%). Acetic acid (99%) was purchased from Sigma-Aldrich. Epinephrine (C_9_H_13_NO_3_), β-estradiol (C_18_H_24_O_2_; ≥98%), 4-chlorophenol (ClC_6_H_4_OH), bisphenol A (C_15_H_16_O_2_; ≥99.9%), gold (III) chloride hydrate (HAuCl_4_), 3,4-ethylenedioxythiophene (EDOT; 97%) hydrochloric acid (HCl), and dibuthyl phthalate (C_16_H_22_O_4_; ≥99%) were purchased from Sigma-Aldrich. All other reagents were of analytical grade and used as received without any pretreatment.

A CO_2_ laser tool (Universal Laser Systems® PLS6.75) was used for laser scribing onto a PI substrate under ambient conditions. The spot diameter and wavelength are fixed as ∼150 and 10.6 μm, respectively. Prior to the graphene production, necessary optimizations were performed following our previous work, and the DPI was set as a constant to a value of 1,000 to maintain the graphene quality (Beduk et al., 2020). The sensor design was prepared by using the L-Edit software v15.0 from Tanner EDA and uploaded to the laser system. Morphological characterization was done by TENEO VS scanning electron microscope (TENEO VS SEM). The x-ray diffraction data were recorded using an x-ray diffractometer (Bruker Corporation, D8 ADVANCE, and Karlsruhe, Germany) with Cu Kα radiation (1.5406 Å) and 2θ range of 20°–80°. Raman data were obtained at 473 nm with a cobalt laser source by using a LabRAM ARAMIS Raman spectrometer (Horiba Scientific). Elemental analysis of LSG surfaces was performed by using x-ray photoelectron spectroscopy (XPS) from Kratos Analytical (AMICUS/ESCA 3400) with an Al-Kα x-ray source (1,468.6 eV) applied at 10 kV that generated 10-mA current. Topological imaging and roughness measurements were performed using the Bruker Dimension Icon AFM system. The electrochemical measurements were performed by both our homemade potentiostat device connected to a smartphone and a commercial electrochemical measurement workstation (Palmsens 4) connected to a computer and controlled by the PSTrace 5.5 software. All experiments were performed in triplicate with LSG reference and counter electrodes at room temperature and at pH 7.4.

### 2.2 Preparation of AuNPs Modified LSG-MIP Sensor

**FIGURE 1:**
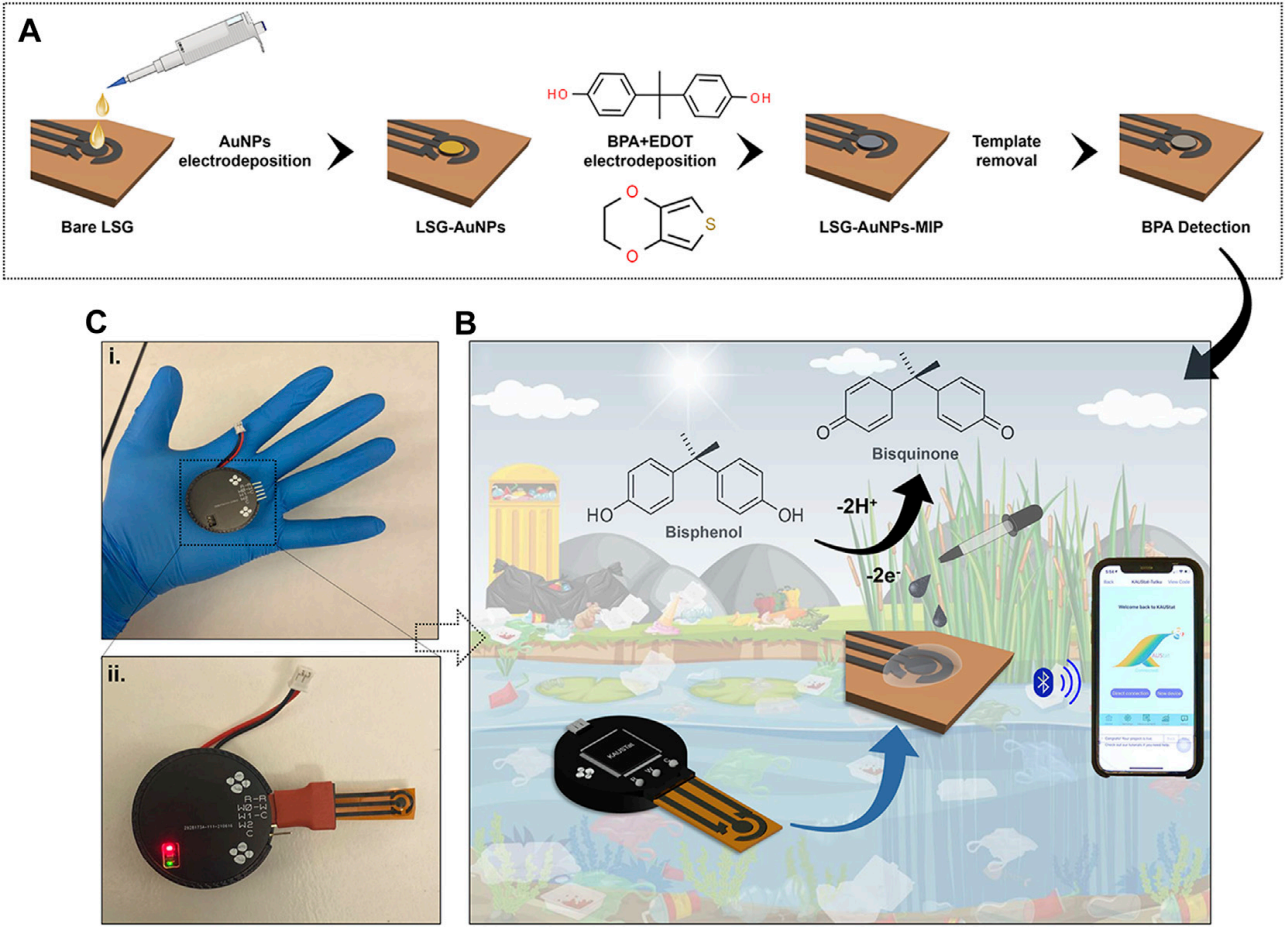
**(A)** Preparation of the molecular imprinted laser-scribed graphene (LSG) sensor including electropolymerization of gold nanoparticles (AuNPs), 3,4-ethylenedioxythiophene (EDOT) monomer in the presence of bisphenol A (BPA), and template removal to create BPA cavities. **(B)** Schematic illustration of the portable potentiostat attached to the AuNPs/LSG sensor and connected to a smartphone via Bluetooth for detecting BPA in environmental samples. **(C)** The pictures of **(i)** the potentiostat, **(ii)** the device combined with the LSG-MIP sensor. Red light indicates an active Bluetooth connection.

Gold chloroauric acid (50 mM) was prepared in 50 mM HCl as the precursor solution for gold (AuNPs) electrodeposition. Of the gold solution, 70 µl was placed onto the LSG working electrode to perform the chronoamperometry method with respect to the LSG reference and counter electrodes. The current was recorded at a fixed potential value of −0.9 V with a time interval of 0.1 s for 270 s, optimized in our previous work (Rauf et al., 2021). The surface was cleaned and dried with N_2_ gas following the AuNP electrodeposition. EDOT monomer was electropolymerized on the working electrode (WE) surface in the presence of 1 mM BPA solution at a fixed potential value of 0.85 V for 70 s. About 10 mM EDOT was mixed with 1 mM BPA in 50 mM PBS at pH 7.4. About 10 mM BPA was prepared in dimethyl sulfoxide:50 mM PBS (3:7, v/v) as the stock solution. Following the adduct step, the attached BPA was removed from the natural cavities on the PEDOT layer by acetic acid:methanol (3:7, v/v) solution for 15 min. The preparation process is schematically described in Figure 1A. As the final step, the empty cavities were filled with different concentrations of BPA for the rebinding step. The difference in the current response was observed depending on the occupancy of the BPA-specific cavities on the WE surface.

For reference purposes, the AuNP-/LSG-modified nonimprinted polymer LSG/AuNP-NIP is prepared by the electropolymerization of EDOT in the absence of BPA. Following the AuNP modification on the surface, the EDOT monomer was electropolymerized on the working electrode surface without BPA presence. The same fixed potential value of 0.85 V and duration were used for the chronoamperometry. EDOT (10 mM) was prepared in 50 mM PBS at pH 7.4.

### 2.3 Electrochemical Measurements

A potential range of −0.6 to +0.6 V at a pulse amplitude of 50 mV and a pulse width of 0.1 s with a scan rate of 100 mV/s was used to carry out the differential pulse voltammetry (DPV) method by a commercial potentiostat. The same potential range was used to carry out cyclic voltammetry (CV) with a scan rate of 100 mV/s. DPVs by KAUSTat were obtained in the potential range from −0.45 to +0.45 V for the rest of the sensing performance investigation.

### 2.4 Development of KAUSTat: A Fully Integrated Home-Made Potentiostat

KAUSTat is a potentiostat device that enables multiple amperometric and voltametric measurement techniques developed in our previous work (Ahmad et al., 2019). Figure 1C shows the handheld potentiostat device connected wirelessly to a smartphone. The device has a Programmable System on Chip (PSoC) 5LP, sensor input/output peripherals, an SD card slot, a Bluetooth module (CYBLE-214015-01), a power management circuit, an RS232-to-USB converter, LEDs, and a micro-USB port for smartphone connection. A customized mobile application software was developed having multiple operation options for connection, parameter, control, and data visualization. The Programmable System on Chip (PSoC) 5LP manages the reconfigurable pins (electrodes) and the internal potentiostat circuitry. Thus, KAUSTat has proven that it has the potential of being an accurate and portable electrochemical detection system, compared with the large-scale commercial electrochemical setups.

### 2.5 Real Sample Preparation

Two different brands of bottled water and tap water samples were selected and spiked with BPA to test the LSG-MIP sensor performance. Each water sample was mixed with 50 mM PBS in 50% ratio and then was used to prepare 0.1 and 1 μM BPA solutions. Then analyte solutions prepared with water samples were incubated on the sensor for 20 min. Following the water samples, two different brands of plastic bottles were tested with and without spiking. Of the plastic pieces, 2 g was mixed with 50 ml of deionized water inside a glass container covered by aluminum foil and kept on a hot plate at 70°C for 16 h. After the solutions were cooled, 5 ml of each plastic solution was mixed with 5 ml of 50 mM PBS to incubate for 20 min at the rebinding step. In addition to directly measuring plastic solutions, they were also spiked with 0.1 and 1 μM BPA to observe the current change at different concentrations of BPA. Finally, two different bottled milk samples and a local brand of baby formula solution was spiked with 0.1 and 1 μM BPA and tested. Of each sample, 5 ml was mixed with 5 ml of 50 mM PBS to prepare the BPA solutions for incubation onto the sensor.

## 3 Results and Discussion

### 3.1 Design of the Portable Potentiostat Device

**FIGURE 2:**
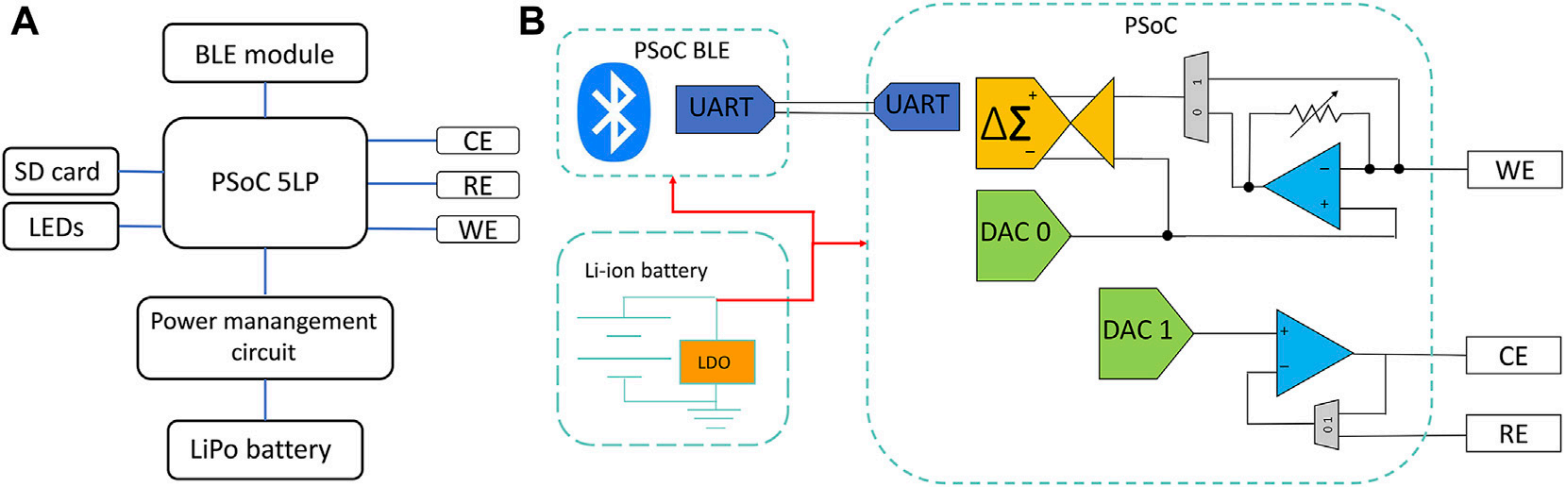
**(A)** Block diagram of the device components. **(B)** Potentiostat circuitry implemented in KAUSTat.

A reconfigurable potenstiostat called KAUSTat was designed in-house to conduct sensing measurements on-site. The device presents a proof of concept for in-field deployment of our sensor. KAUSTat is suitable for reprogramming of the internal circuitry according to the requirements of a specific application. Similar to an FPGA (field-programmable gate array), a design was created and deployed into the KAUSTat core. The main circuitry consists of a 32-bit Arm® Cortex®-M3 programmable system on chip (PSoC), a Bluetooth module (CYBLE-214015-01) and a 3.7-V, 70-mA/h lithium battery. This design consists of a standard potentiostat circuitry having three electrodes and embodies a delta–sigma analog-to-digital converter (∆∑-ADC), two digital-to-analog converters (DAC), and two operational amplifiers with programmable feedback resistance. The device can also change its impedance to control the current flow into the electrode. This circuitry modification happens directly in the operational amplifier and feedback resistance, complementary to the voltage potential applied into the electrodes. The optimization of the device including the impedance effect was investigated in our previous work (Beduk et al., 2021b). Two multiplexes were added to the system to allow the potential of the sensor readout change from three- to two-electrode cells. The side and top views of the device are presented in Supplementary Figure S1. The total diameter and the height of the device were measured as 4 and 1.5 cm, respectively. The device weighs 20 g with the battery and case. A user-friendly mobile application was developed to control the electrochemical techniques applied by KAUSTat. Multiple windows are available at the interface for control, method selection, operation, and data visualization, shown in Supplementary Figure S2. The communication between the smartphone and KAUSTat is made possible through the Generic Attribute Profile (GATT) Bluetooth protocol. Universal asynchronous receiver/transmitter (UART) was established to communicate with the PSoC. Figure 2 shows the block diagram of the peripherals and circuitry of the device.

### 3.2 Characterization of LSG-MIP and LSG-NIP Sensor

**FIGURE 3:**
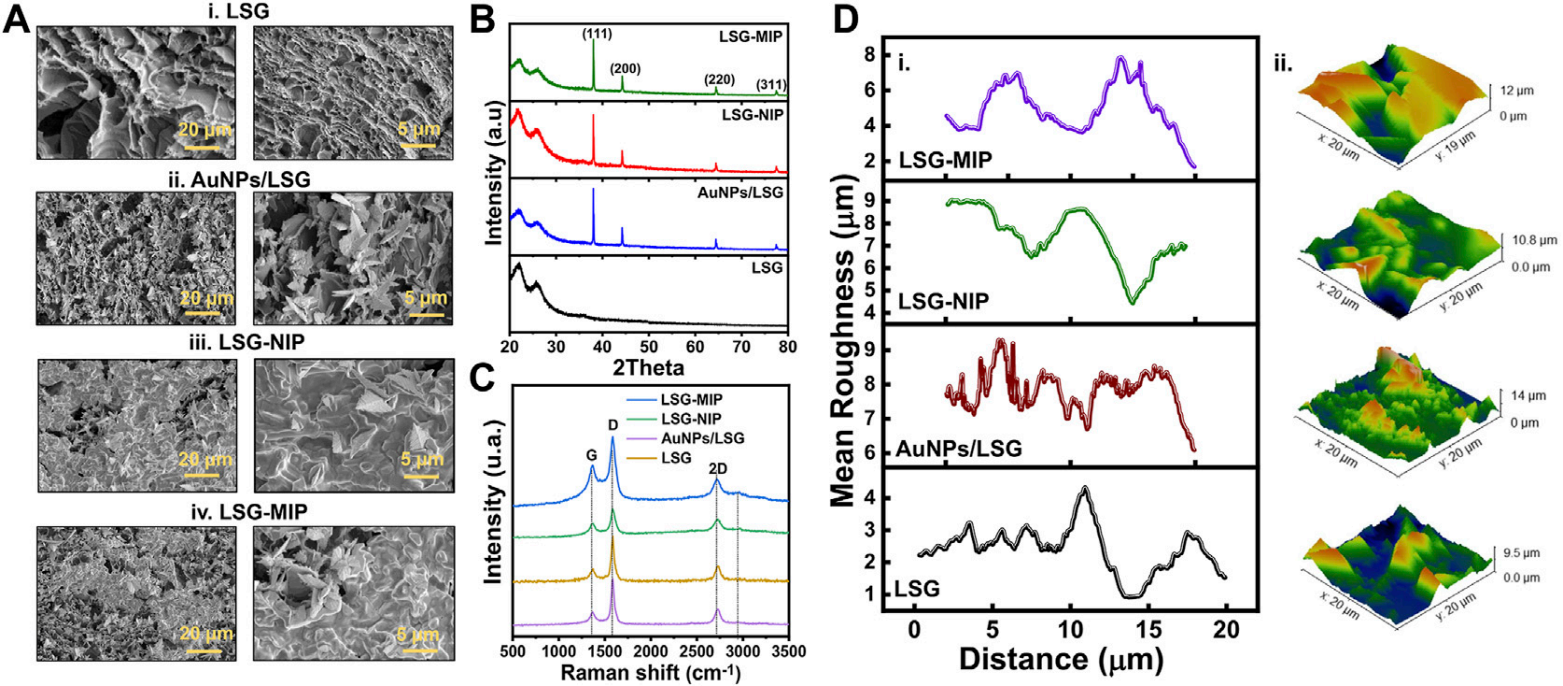
**(A)** SEM images with scale bars of 20 and 5 µm, **(B)** XRD spectra, **(C)** Raman spectra, and **(D)** AFM mean roughness measurements and 3D topology images of **(i)** bare LSG, **(ii)** LSG/AuNPs, **(iii)** LSG-NIP (LSG-AuNP-PEDOT), **(iv)**. LSG-molecularly imprinted polymer (MIP) (LSG-AuNP-PEDOT-BPA) sensor.

The representative SEM images in Figure 3A show the difference in structure for bare LSG, AuNP/LSG, LSG-NIP, and LSG-MIP. The gold nanoparticle flakes are clearly observed in alignment of our previous work (Rauf et al., 2021). Following the EDOT polymerization, the LSG flakes were observed as covered by the PEDOT layer. The gold flakes are embedded in the polymer matrix in LSG-NIP and LSG-MIP samples. Results were supported by EDX analysis shown in Supplementary Figure S3 including mapping. The presence of carbon, oxygen, nitrogen and sulfur was confirmed after the deposition of the PEDOT layer. In addition to qualitatively identifying the elements in each sample, XPS analysis was conducted for each sample. The high-resolution spectra of each sample are displayed in Supplementary Figure S4. The C 1s high-resolution spectrum shows the characteristic peaks of the C=C bonds of graphene. In addition, the peaks located at 85.3 and 89.9 eV were the characteristic peaks of Au 4f_7/2_ and Au 4f_5/2_ belonging to the AuNPs electrodeposited onto graphene. Following EDOT polymerization, N–C (sp^3^) bonding occurs on the surface, visible in N 1s high-resolution spectra (Vusa et al., 2017). Broad peaks of S 2p spectrum and relatively sharp peaks of C1s spectrum represent S 2p_1/2_ and C–C, C–S, C–O–C, C=O bondings, respectively (Saxena et al., 2011; Abas et al., 2019). Supplementary Table S1 has mass percentage values of LSG, AuNP/LSG, LSG-MIP, and LSG-MIP sensor surface. This quantitative analysis proved the presence of N 1s, S 2p, as well as the increase in C 1s, O 1s composition in NIP and MIP samples. The surface of the LSG-NIP electrode is observed as having the highest composition of S, proving the PEDOT polymer layer coverage.

In contrast, the LSG-MIP surface, having both PEDOT layer and BPA-filled cavities created in the polymer matrix, exhibits lower S and higher C, O composition. In addition to elemental characterization, crystallinity of the sensor surface was investigated. XRD spectra in Figure 3B indicates two clear peaks at 2θ = ∼21.3° and ∼25.6°, indicating the (002) plane coming from the high degree of graphitization (Beduk et al., 2020). After gold electrodeposition, the XRD spectrum exhibited a high degree of crystallinity at 38.01°(1 1 1), 44.18°(2 0 0), 64.48°(2 2 0), and 77.37°(3 1 1) indicating the gold coverage onto the LSG surface, matching with the ICSD reference code of 96-900-8464 (Krishnamurthy et al., 2014). Further polymer modification onto the gold surface does not affect the crystallinity as observed in Figure 3B. The Raman spectra in Figure 3C shows the highest I2D/IG peak intensity ratio in bare LSG correlating with the presence of graphene. The mean roughness profiles of the working electrode surface are presented in Figure 3D for LSG, AuNP/LSG, LSG-NIP, and LSG-MIP. 3D AFM images of samples shows that LSG surface roughness increases after the gold deposition. Following the polymer deposition, surface height significantly increases due to the relatively tick polymer matrix forming on the surface.

### 3.3 Optimization of Experimental Conditions

**FIGURE 4:**
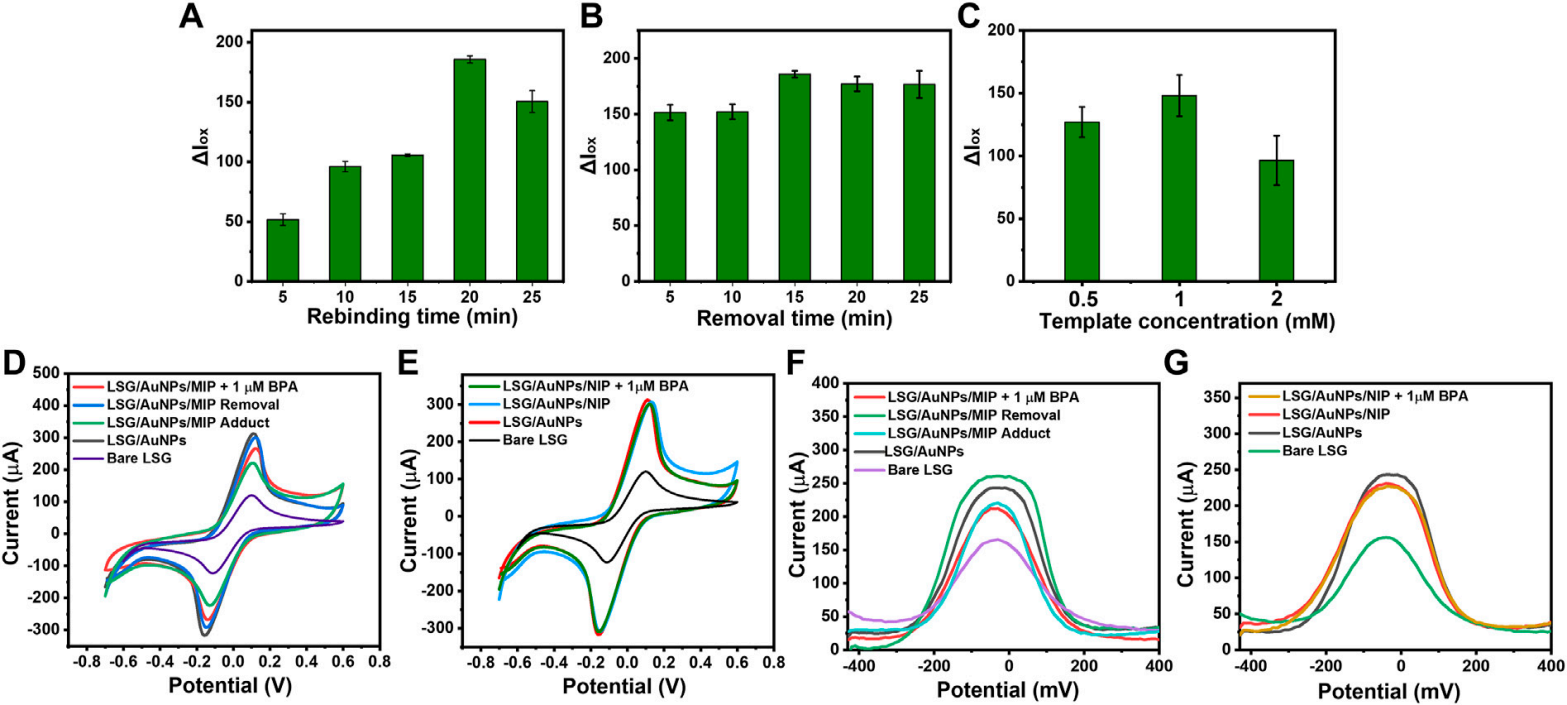
Histograms showing the oxidation current difference before and after rebinding BPA (ΔIox) obtained from differential pulse voltammetries (DPVs) of LSG-MIP for **(A)** rebinding time optimization after binding 1 µM BPA of different rebinding times, **(B)** removal time optimization using 20-min rebinding time for 1 µM BPA, **(C)** template concentration optimization using 15 min of removal time and 20 min of rebinding time for 1 µM BPA. Cyclic voltammetries (CVs) of **(D)** LSG-MIP and **(E)** LSG-NIP in a solution of 5 mM [Fe(CN)_6_]^3−/4−^ with 0.1 M KCl (scan rate: 100 mV/s) performed by Palmsens showing the oxidation response of bare LSG, LSG/AuNP, before and after rebinding of 1 µM BPA. DPVs of **(D)** LSG-MIP and **(E)** LSG-NIP in a solution of 5 mM [Fe(CN)_6_]^3−/4−^ with 0.1 M KCl performed by KAUSTat showing the oxidation response of bare LSG, AuNPs/LSG, before and after rebinding of 1 µM BPA.

Molecular imprinting was performed by electrodepositing EDOT on the surface of the AuNP/LSG. The electrodeposition process took place in the presence of a certain amount of BPA as the template molecule. When the specific cavities formed on the polymer matrix, BPA was removed from the surface. These empty BPA cavities later bind selectively to BPA molecules within a solution, causing an oxidation current difference in electrochemical signal. The polymer layer having embedded BPA cavities interacts with the LSG surface by the hydrogen bonding and the π stacking interactions (Zheng et al., 2018). Prior to electrochemical characterization, we performed optimization for the experimental steps. Rebinding and removal time was optimized by using 1 mM BPA template concentration with a 50 mM EDOT solution. A drop in current response was observed after 20 min of rebinding BPA. This might be due to the over blockage of the current by a high amount of analytes accumulating on the surface. In addition, the removal duration was optimized by removing the template from 5 to 25 min. No significant current change was observed after 15 min of removal time. Therefore, these values were chosen as the optimum, shown in Figures 4A, B. As the final step, template concentration was optimized by keeping 15 min of removal and 20 min of rebinding time constant. Among the three concentrations shown in Figure 4C, the highest oxidation current was obtained from the sensor prepared with 1 mM BPA as the template concentration.

CV results in Figures 4D, E represent the electrochemical characterization performed by a commercial potentiostat. MIP formation, successful removal, and rebinding of the template were observed compared with NIP. DPV response of LSG-MIP and LSG-NIP obtained by KAUSTat is given in Figures 4F, G. Following the gold electrodeposition, a current increase was observed due to the high conductivity and large surface area accelerating the electron transfer. When the EDOT monomer polymerized on the surface to create the MIP matrix, the current decreased due to the presence of the template molecule. Removal procedure empties the BPA cavities eventually leading to a high electroactive area and current intensity. The empty cavities are actively filled once a BPA solution was introduced to the sensor causing a current decrease depending on the analyte concentration. The current responses obtained from both commercial potentiostat and KAUSTat follow the same trend allowing the identification of the BPA concentration by the LSG-MIP sensor. However, the LSG-NIP sensor cannot detect BPA in any concentration due to the absence of analyte-specific cavities.

To investigate the template recognition ability more precisely, the imprinting factor was calculated from the oxidation current responses of the LSG-MIP and LSG-NIP using the following equation:

α=Δ I ox (LSG−MIP) /Δ I ox (LSG−NIP)

where α is the imprinting factor, Δ I _ox_ (LSG-MIP) is the oxidation current difference occurring at the LSG-MIP sensor in the presence of 1 μM of BPA, and Δ I _ox_ (LSG-NIP) is the oxidation current difference occurring at the LSG-NIP sensor in the presence of 1 μM of BPA. The imprinting factor refers to the oxidation response value of the LSG-MIP surface compared with the response value of LSG-NIP to the presence of the same amount of BPA. The imprinting factor was calculated as 9.6, supporting the fact that LSG-MIP has a significantly high absorption capacity toward the specific analyte.

### 3.4 Electrochemical Activity of Bisphenol A on Molecularly Imprinted Polymer Sensor

**FIGURE 5:**
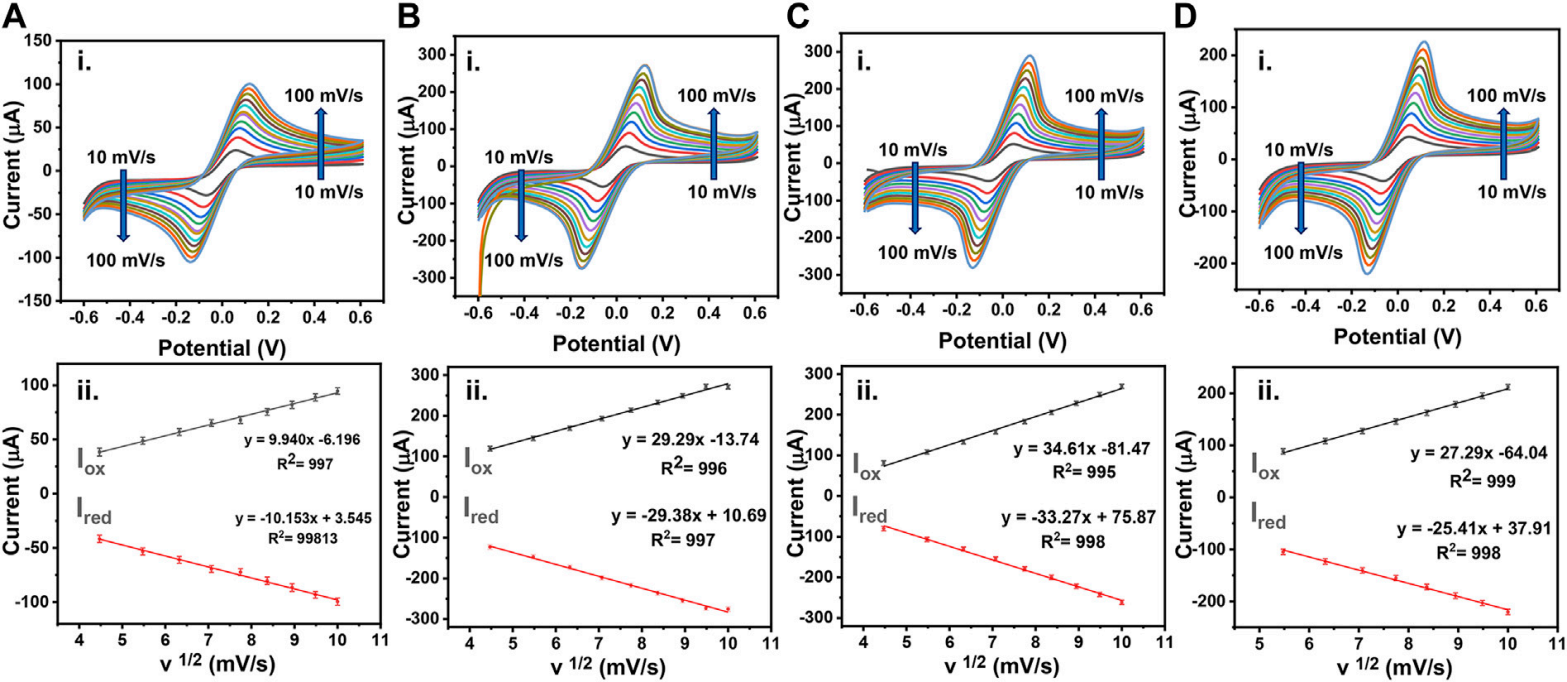
**(i)** CVs obtained using 0.1 M KCl containing 5 mM [Fe(CN)_6_ ]^3−/4−^ as a redox probe or **(A)** bare LSG, **(B)** AuNPs/LSG, **(C)** LSG-NIP, **(D)** LSG-MIP different scan rates from 0.1 to 1 V/s. **(ii)** The plots show I vs. v^½^ (mV/s) for **(A)** bare LSG, **(B)** AuNPs/LSG, **(C)** LSG-NIP, **(D)** LSG-MIP sensor.

The voltametric sweeping was performed for bare LSG electrode, AuNP/LSG, LSG-NIP, and LSG-MIP electrodes in the potential range from −0.6 to 0.6 V. The oxidation and reduction current intensities were recorded at scan rates between 10 and 1 mV/s with respect to bare LSG reference and counter electrodes, shown in Figure 5. The electrochemically active surface area of the sensors was calculated by using the equation below following the Randles–Sevick equation:

Ipa=268600n^(3/2) AD^(0.5)Cv^(0.5)

where *D* is the diffusion coefficient (6.70 × 10^−6^ cm^2^ s^−1^), *A* is the active surface area of the electrode, *C* is the concentration of the redox probe (mol·cm^−3^), *v* is the scan rate (mV s^−1^), and *I*_p_ is the anodic peak current (A). A high conductivity of AuNPs/LSG leads to a higher active surface area (0.143 cm^2^) compared with a bare LSG working electrode (0.080 cm^2^). The redox current values are recorded for AuNP/LSG compared with the bare LSG in redox probe solutions. Supplementary Table S2 summarizes the anodic and cathodic peak values in both [Ru (NH_3_)_6_]^3+^ and [Fe (CN)_6_]^3−^. The variations between measurements are small and provide a repeatable reaction, having enhanced *I*_pa_ and *I*_pc_ values for metal-deposited LSG surface. Figures 5C, D represent the CV response of LSG-MIP and LSG-NIP recorded in [Fe (CN)_6_]^3/4−^ redox probe at the potential range from −0.6 to 0.6 V, respectively. The gold surface was modified with the PEDOT layer in the presence of the template analyte leading to a decrease in electroactivity and an active surface area value of 0.127 cm^2^. On the other hand, the LSG-NIP sensor leads to a higher active surface area (0.162 cm^2^) due to the absence of any template. Equations of corresponding oxidation and reduction CVs are given in Supplementary Table S3. A linear behavior for *I*_p_ vs. the square root of *v* is obtained for each sensor, which indicates a diffusion-controlled charge transfer.

### 3.5 Sensing Performance of Molecularly Imprinted Polymer Sensor

**FIGURE 6:**
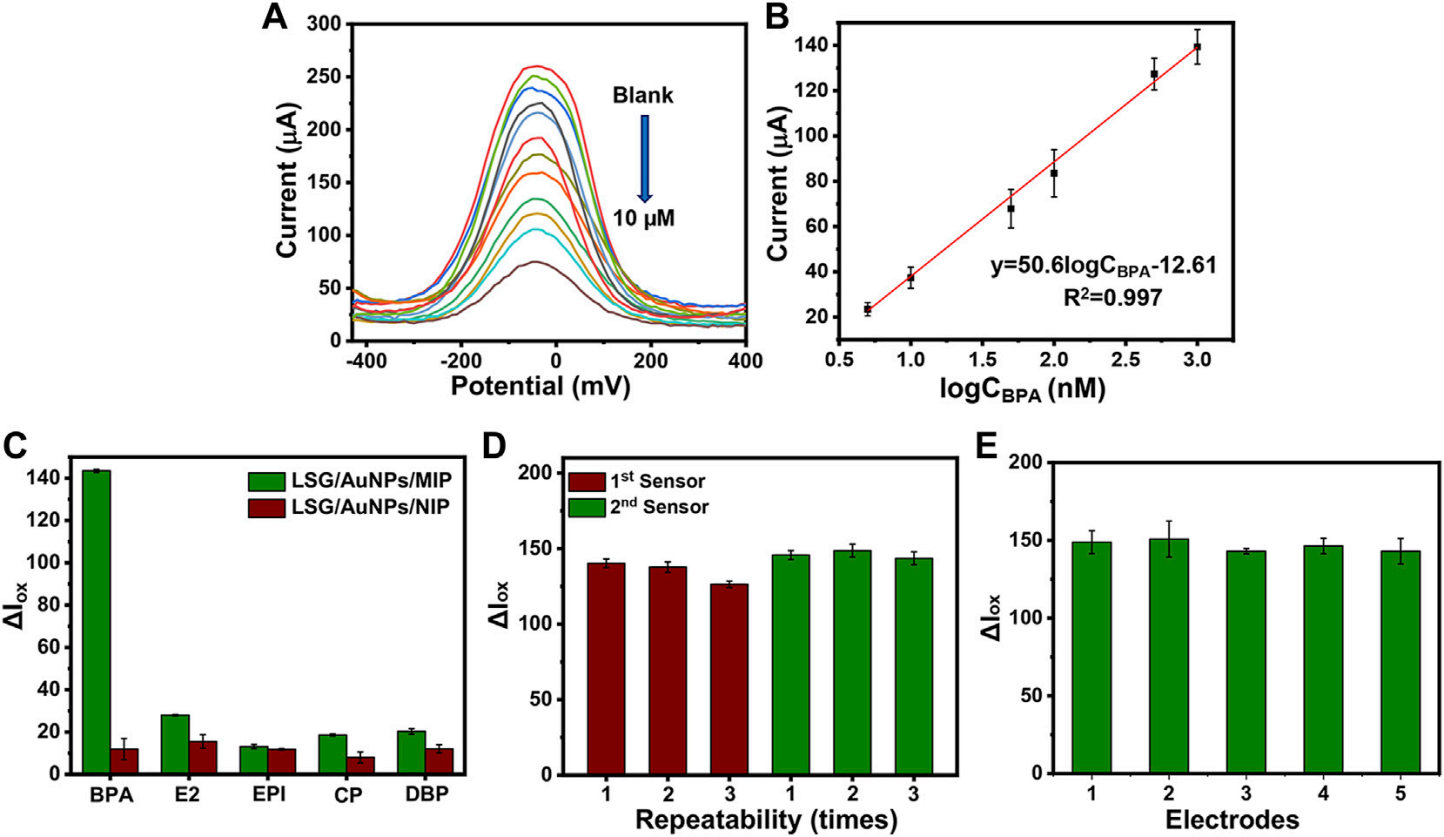
**(A)** DPVs obtained at LSG-MIP incubated in different concentrations of BPA. **(B)** The corresponding calibration plot obtained for LSG-MIP. Histograms obtained from Iox values of **(C)** LSG-MIP and LSG-NIP in the presence of BPA, epinephrine (EPI), β-estradiol (E2), 4-chlorophenol (CP), and dibutyl phthalate (DBP). The concentration of BPA and other inferences used was 1 μM, **(D)** LSG-MIP showing the reusability of two different sensors performing removal and rebinding three times consecutively. **(E)** Reproducibility test showing the response of five different LSG-MIP sensors prepared with the same procedure. The measurements were done by using optimal conditions (1 mM BPA, .5 M EDOT, 15 min of removal time, and 20 min of incubation time of 1 μM of BPA). KCl (0.1 M) containing 5 mM [Fe(CN)_6_ ]^3−/4−^ was used as the redox probe.

The oxidation current response of the LSG-MIP sensor was recorded after rebinding different concentrations of BPA by the portable KAUSTat device. The calibration curve on Figure 6B shows a direct relation between the current response and logC_BPA_. High concentrations of the analyte occupy the electroactive imprinted cavities more, leading to a poor conductivity on the surface. The equation obtained from the calibration curve was ΔI (μA) = 50.6logC_BPA_ −12.61 (R= 0.997). The detection limit (LOD) was calculated as 3.97 nM from a linear range of 0.01–10 µM (LOD= 3б/S, S/N= 3). In addition to KAUSTat, a commercial potentiostat was also used to validate the performance of the LSG-MIP sensor. The LOD value was calculated as 15 nM in the range of 0.01–10 µM from the calibration equation of ΔI (μA) = 47.1logC_BPA_ + 26.31 (R^2^= 0.997), shown in Supplementary Figure S5. Since the enhanced sensitivity was obtained by the portable potentiostat device, the rest of the sensing performance tests were conducted by KAUSTat. In another sensing performance test, selectivity was tested by recording the electrochemical responses of the other interferences by KAUSTat having structural similarities with the main analyte, estradiol, epinephrine, dibutyl phthalate, and 4-chlorophenol examined by the same procedure with the previously optimized parameters for BPA. The LSG-MIP sensor responded slightly to 1 µM of the interferences shown in Figure 6C. However, an approximately fourfold increase was observed in the response of BPA compared with the current response of interferences, confirming the high affinity of the LSG-MIP sensor toward BPA. On the other hand, the LSG-NIP sensor did not particularly show a significant result for any of the compounds including BPA due to the lack of cavities and specific binding. The high selectivity is due to the imprinted polymeric matrix specifically designed and optimized for the BPA molecule. The conditions of MIP synthesis, such as selection of appropriate functional monomer, removal time and agent, and incubation time were optimized among various parameters, resulting in a customized synthesis method for MIP toward the detection of BPA on an LSG sensor.

The possibility of reperforming the removal procedure for the same sample was investigated to understand the reusability of the same sensor. Figure 6D shows the oxidation current responses of two different sensors with three removal–rebinding cycles. After every cycle, the sensor response is slightly reduced. Finally, the 10% of the initial sensor response faded after three removal–rebinding cycles. This current loss can be explained by the slight damage coming from using an acetic acid/methanol (3:7) mixture as the removal solution to remove BPA from the previously created cavities. In addition, Figure 6E shows the current responses coming from five different LSG-MIP sensors prepared by the exact same method. Having approximately 94.8% of the same current value stable for each electrode as the response to 1 μM BPA explains that the proposed imprinting method provides a stable alternative to the existing BPA sensors in the market.

### 3.6 Determination of Bisphenol A in Real Samples

The verification of the LSG-MIP sensor performance was performed by using real samples shown in Table 1. Two local brands of bottled water and milk samples, tap water, and a local brand of baby formula solution were spiked with a known amount of BPA to obtain recovery values. By spiking samples by 0.01 and 1 μM BPA, calculated RSD (%) values are observed as varying between 82.84% and 120.2%. In addition to the liquid samples, two local brands of bottled water were used to detect BPA. Following a consistence heating procedure, the initial concentration of BPA was detected along with the recovery values of the spiked amounts. Recovery values ranging between 97.27% and 112.2% proves the successful detection of BPA at varying concentrations in real samples.

**TABLE 1:** Determination of BPA in water, milk and plastic samples by LSG-MIP sensor.

	Commercial potentiostat					KAUSTat				
	Initial concentration (nM)	Added (nM)	Found (nM)	Recovery (%)	RSD	Initial concentration (nM)	Added (nM)	Found (nM)	Recovery (%)	RSD
Water sample 1	0	10	8.695	86.95	9.88	0	10	12.024	120.2	1.23
	0	1,000	1179.3	117.9	3.12	0	1,000	828.42	82.84	3.69
Water sample 2	0	10	15.917	159.2	9.87	0	10	10.417	104.174699	8.68
	0	1,000	939.69	93.96	1.81	0	1,000	1,093.1	109.3	.68
Tap water	0	10	7.667	76.67	4.04	0	10	9.339	93.39	8.87
	0	1,000	983.03	98.30	9.88	0	1,000	1,069.6	106.9	1.06
Milk sample 1	0	10	11.059	110.6	1.99	0	10	9.1967	91.97	8.79
	0	1,000	941.05	94.11	3.93	0	1,000	1,092.8	109.3	1.98
Milk sample 2	0	10	14.114	141.1	3.85	0	10	11.08	110.8	3.45
	0	1,000	799.73	79.97	8.04	0	1,000	1,034.7	103.4	1.98
Baby formula	0	10	10.616	106.2	3.83	0	10	11.701	117.0	6.26
	0	1,000	1,029.9	102.9	4.31	0	1,000	1,057.9	105.8	6.23
Plastic bottle 1	344.45	100	397.12	86.26	1.45	119.43	100	126.2	105.7	1.78
	344.45	1,000	1,348.1	106.5	3.36	133.23	1,000	1,134.0	112.2	5.41
Plastic bottle 2	302.44	100	421.97	101.1	0.48	119.43	100	140.13	97.27	0.79
	302.44	1,000	1,311.8	103.1	3.54	133.2	1,000	1,129.6	105.2	6.31

## 4 Conclusion

Custom-made LSG-MIP sensors were manufactured using standard laser irradiation and electrodeposition method to create a synthetic receptor matrix. Having AuNPs coupled with the PEDOT matrix enhances the stability of the platform and the sensitivity toward BPA in terms of the electrochemical response. The formation of the electrodeposited layers was corroborated by morphological and elemental characterization methods. The electrochemical performance and the active surface area of the developed sensor were characterized by cyclic voltammetry using both [Fe(CN)_6_]^3−^ and [Ru(NH_3_)_6_]^3+^ redox probes. The electrochemically active surface area was found to be 30% lower for LSG-MIP compared with LSG-NIP due to the occupation of the cavities with the template molecule. The developed sensor was combined with a portable potentiostat connected to a smartphone via Bluetooth. Having a customized smartphone app, our homemade potentiostat does not require any particular training to operate and is fully applicable to on-site pollutant detection without the need of any bulky detection system. In this study, the practicality allows users to monitor BPA existence in environmental samples on-site. This customized device exhibited relatively higher selectivity and sensitivity (LOD: 3.97 nM) compared with laboratory-based potentiostat systems, proving its great prominent potential as an electrochemical transducer for chemical and bioassays. This new sensing platform could be easily extended to monitor the levels of other endocrines disrupting chemicals in complicated matrices.

## Data Availability

The original contributions presented in the study are included in the article/Supplementary Material, Further inquiries can be directed to the corresponding authors.
