# Supplementary material for: A Portable Molecularly Imprinted Sensor for On-Site and Wireless Environmental Bisphenol A Monitoring
Source: Front Chem. 2022 Feb 16;10:833899. doi: 10.3389/fchem.2022.833899 (PMC8888969; doi:10.3389/fchem.2022.833899)
Supplement: Supplementary file 1 [file DataSheet1.docx]

Supplementary Material

**TO**

**A portable molecularly imprinted sensor for on-site and wireless environmental Bisphenol A monitoring**

Tutku Beduk^1^, Matilde Gomes^1^, José Ilton de Oliveira Filho^1^, Saptami Suresh Shetty^1^, Walaa Khushaim^1^, Ricardo Garcia-Ramirez^1^, Ceren Durmus^1^, Abdellatif Ait Lahcen^1^*, Khaled Nabil Salama^1^*

^a^Sensors Lab, Advanced Membranes and Porous Materials Center (AMPMC), Computer, Electrical and Mathematical Science and Engineering (CEMSE) Division, King Abdullah University of Science and Technology (KAUST), Thuwal 23955-6900, Saudi Arabia

*Corresponding authors: [khaled.salama@kaust.edu.sa](mailto:khaled.salama@kaust.edu.sa); [abdellatif.aitlahcen@kaust.edu.sa](mailto:abdellatif.aitlahcen@kaust.edu.sa).

# KAUSTat hardware

#
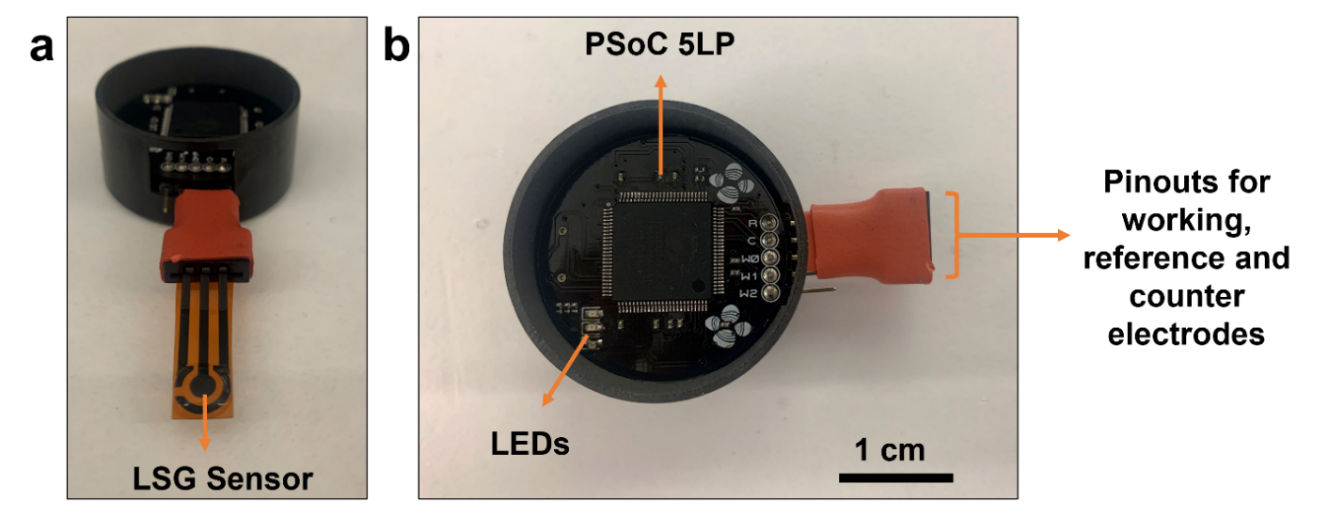


# Supplementary Figure 1. a) Side view and b) top view of the device showing the components.


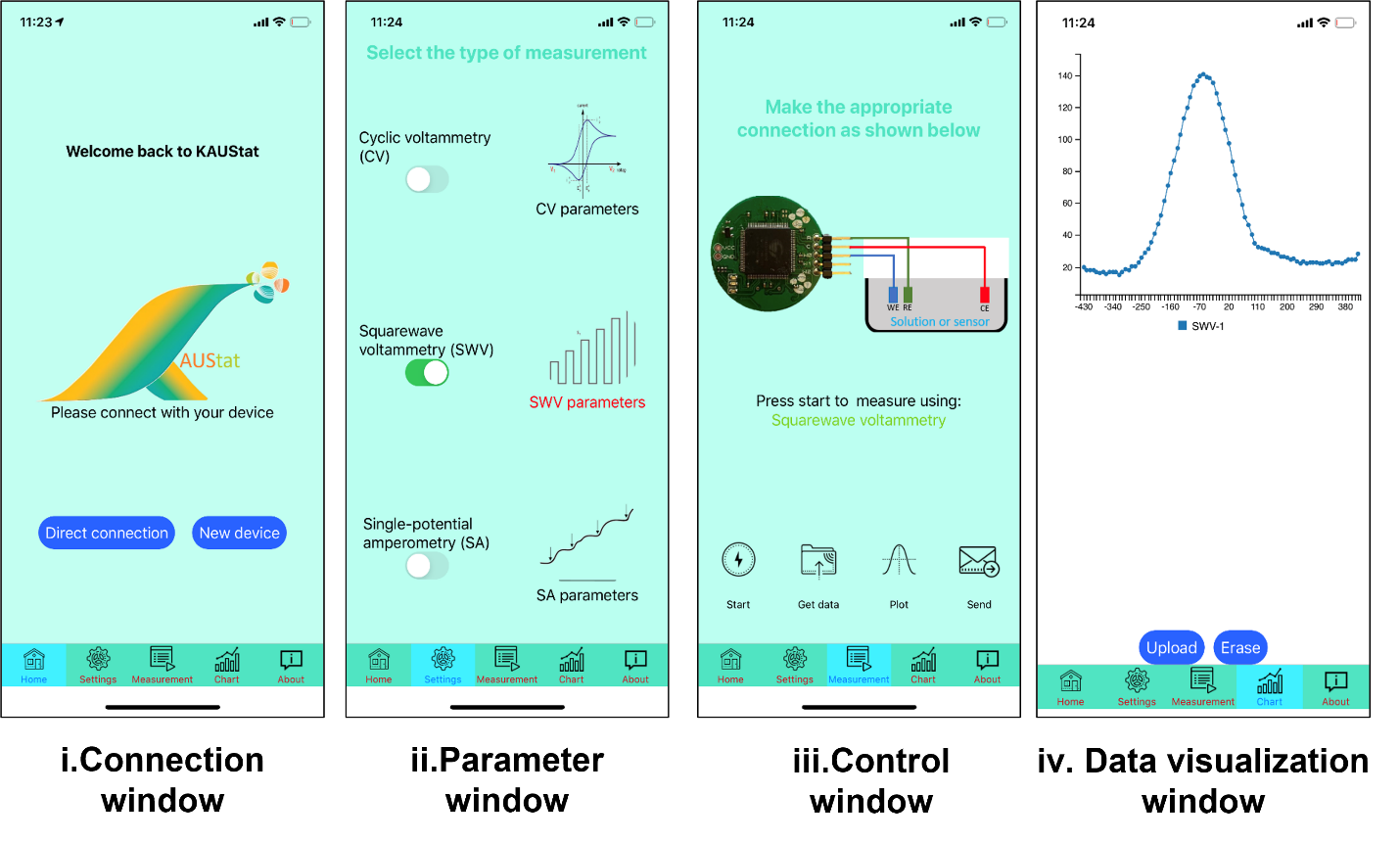


Supplementary Figure 2. The software interface, having i. Connection, ii. Parameter, iii. Control and iv. Data visualization windows.

# Characterizations


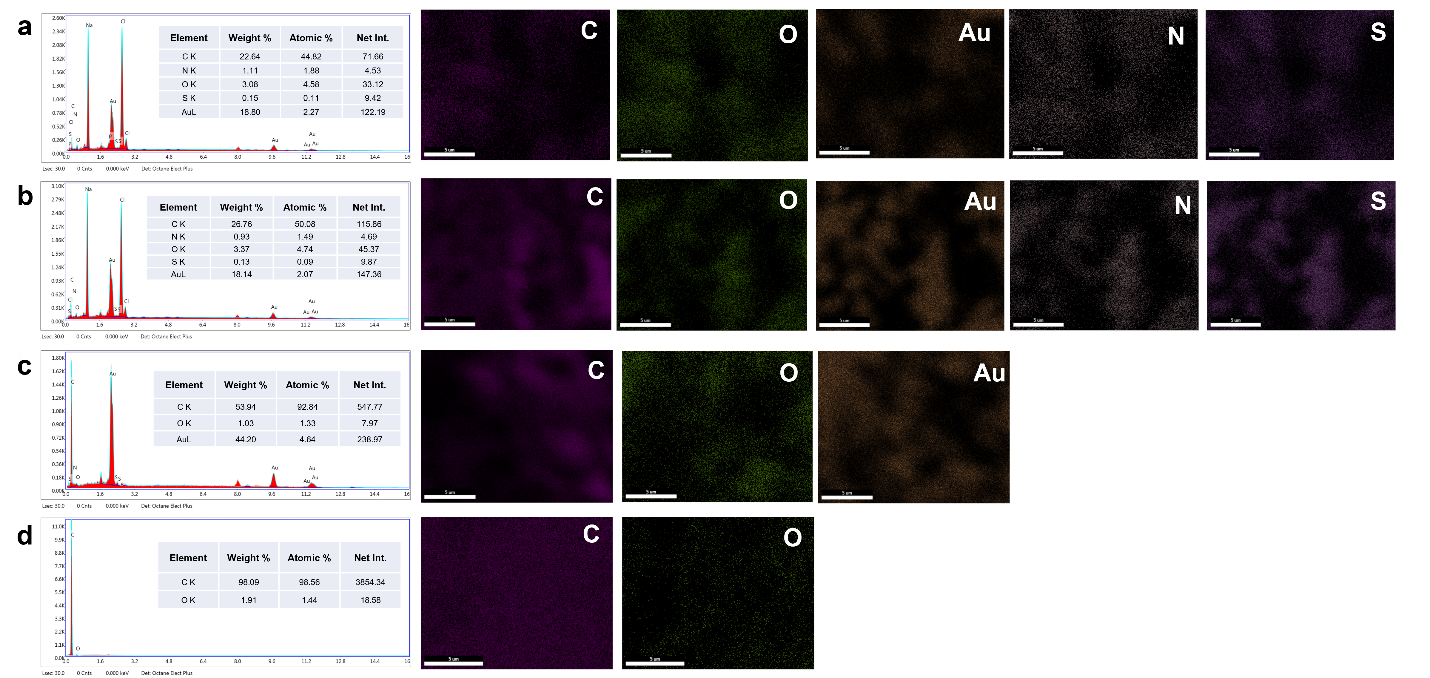


Supplementary Figure 3. Elemental composition of a) LSG-MIP, b) NIP-LSG, c) AuNPs/LSG, d) Bare LSG electrode surface with energy Dispersive X-Ray Analysis (EDX) mapping images (scale bar: 5 µm).


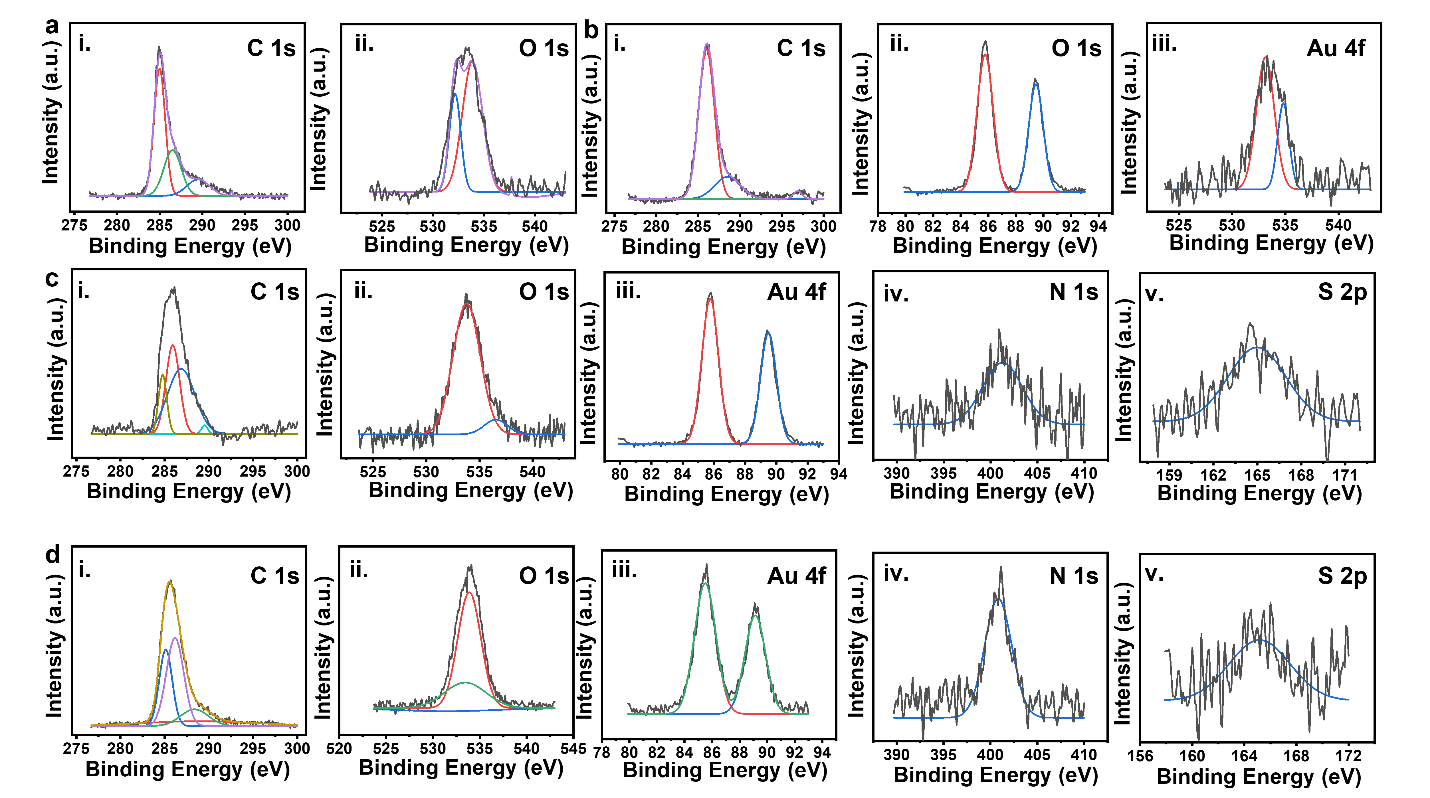


Supplementary Figure 4. X-ray photoelectron spectroscopy (XPS) data containing C1s, O 1s, N 1s, S 2p, and Au 4f7 spectra of a) Bare LSG (i-ii), b) AuNPs/LSG (i-iii), c) LSG-NIP (i-v), d) LSG-MIP (i-v) sensor.

Supplementary Table 1. Mass percentage values of LSG, AuNPs/LSG, LSG-NIP and LSG-MIP sensor surface obtained from XPS analysis.

| Mass % | | | | |
| --- | --- | --- | --- | --- |
|  | **LSG** | **AuNPs/LSG** | **AuNPs/LSG-NIP** | **AuNPs/LSG-MIP** |
| **C 1s** | 86.52 | 65.64 | 48.62 | 69.10 |
| **O 1s** | 10.78 | 2.37 | 7.39 | 14.53 |
| **Au 2f** | - | 31.98 | 23.85 | 3.59 |
| **N 1s** | 2.70 | - | 9.68 | 9.89 |
| **S 2p** | - | - | 10.46 | 2.88 |

Supplementary Table 2. Electrochemical Performance Metrics of Bare LSG and AuNPs/LSG electrodes with two different redox probes.

| Electrode | Electrode Number | Redox probe | I_pa_ (μA) | I_pc_ (μA) | I_pa_/I_pc_ (a.u.) | E_pa_ (V) | E_pc_ (V) | ΔE_p_ (V) |
| --- | --- | --- | --- | --- | --- | --- | --- | --- |
| LSG | 1 | [Fe(CN)_6_]^3−^ | 101.32 | -109.21 | 0.96 | 0.11 | -0.12 | 0.23 |
|  |  | [Ru(NH_3_)_6_]^3+^ | 114.96 | -124.36 | 0.85 | 0.03 | -0.30 | 0.33 |
|  | 2 | [Fe(CN)_6_]^3−^ | 117.68 | -125.50 | 0.94 | 0.10 | -0.10 | 0.20 |
|  |  | [Ru(NH_3_)_6_]^3+^ | 120.35 | -121.93 | 0.75 | -0.20 | -0.53 | 0.33 |
| AuNPs/LSG | 1 | [Fe(CN)_6_]^3−^ | 163.09 | -159.13 | 1.02 | 0.11 | -0.11 | 0.22 |
|  |  | [Ru(NH_3_)_6_]^3+^ | 152.21 | -183.09 | 0.83 | -0.27 | -0.55 | 0.28 |
|  | 2 | [Fe(CN)_6_]^3−^ | 169.60 | -167.11 | 1.01 | 0.14 | -0.14 | 0.28 |
|  |  | [Ru(NH_3_)_6_]^3+^ | 167.42 | -209.42 | 0.80 | -0.28 | -0.64 | 0.36 |

# Active surface area calculations

Supplementary Table 3. Randles-Sevick equations LSG electrodes prepared by KAUSTat based on CV measurements in 0.05 M [Fe (CN)_6_]^3-/4-^ and 0.1 M KCl.

| **Electrodes** | **Oxidation** | **Reduction** |
| --- | --- | --- |
| **Bare LSG** | Ipa = 9.94 v^1/2^ – 6.196 | Ipa = 10.153 v^1/2^ + 3.545 |
| **AuNPs/LSG** | Ipa = 29.29 v^1/2^ – 13.74 | Ipa = 29.38 v^1/2^ + 10.69 |
| **LSG-NIP** | Ipa = 34.61 v^1/2^ – 81.47 | Ipa = 33.27 v^1/2^ + 75.87 |
| **LSG-MIP** | Ipa = 27.29 v^1/2^ – 64.04 | Ipa = 25.41 v^1/2^  + 37.91 |

# Calibration curve by Commercial Potentiostat


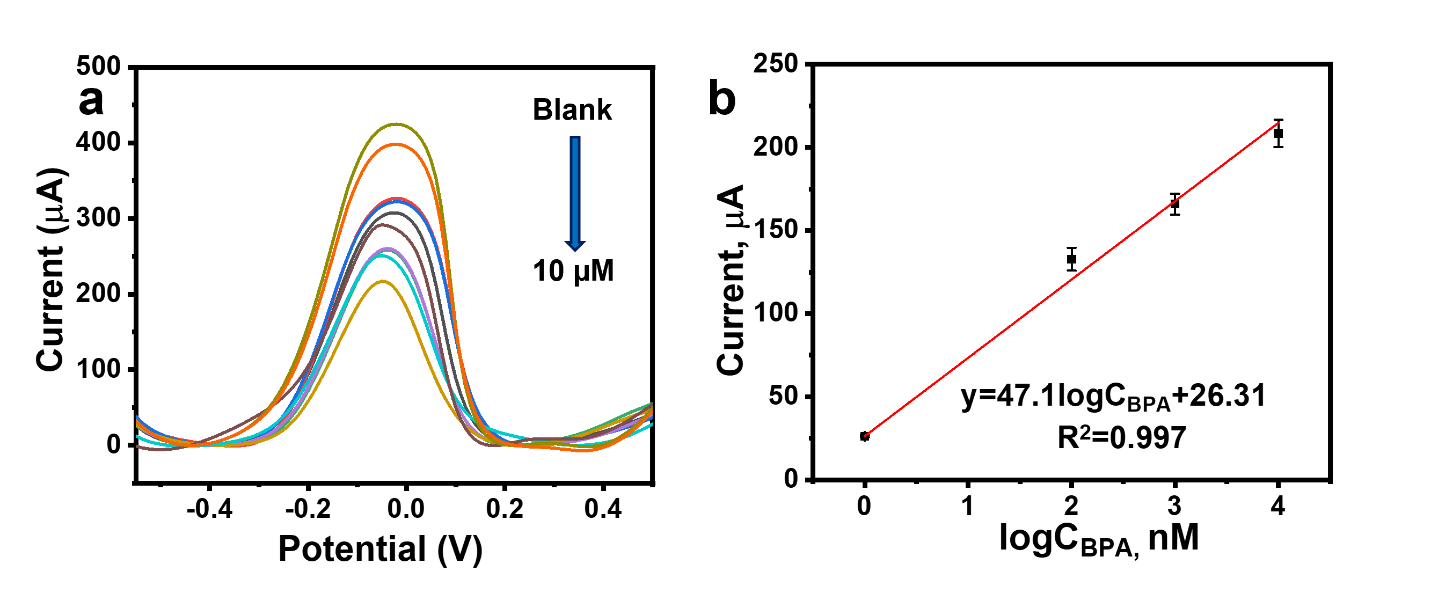


Supplementary Figure 5. a) Differential pulse voltammogram responses of LSG-MIP for different concentrations of BPA in 0.05 M [Fe (CN)_6_]^3-/4-^ and 0.1 M KCl at pH 7.4. b) Calibration curve obtained in the range of 0.05-10 μM.

Supplementary Table 4. Comparison of the developed sensing system with other previously reported BPA sensors.

| Sensing System | Electrochemical method | Linear range/μM | Detection limit/nM | Real Sample |
| --- | --- | --- | --- | --- |
| MIP/mGCE (Zhang et al., 2020a) | CV/EIS | 0.002-5×10^3^µgL^-1^ | 2×10^-4^ µgL^-1^ | Sea water and fish sample |
| MIP/AuNPs/GCE (Jebril et al., 2021) | CV | 8–6 × 10^4^ | 138 | Tap and mineral water |
| MIP/MWCNT (Anirudhan et al., 2018) | CV/DPV | 400-100 | 0.02 | Baby bottles |
| MIP/AuNPs/CBNPs/SPCE (Ben Messaoud et al., 2018) | DPV | 0.07–10 | 8.8 | Tap and mineral water |
| Double oriented MWCNT(Zhang et al., 2020c) | CV/DPV | 0.0001-10 | 0.0157 | Plastic |
| MIP/g-C_3_N_4_r/FTO (Yan et al., 2018) | DPV | 5−100 | 1300 | Bottled water |
| MIP/AgNPs (Wang et al., 2018) | SERS | 100-10 | 50 | Polycarbonate plastic |
| MIP/Pt (Zheng et al., 2018) | CV/DPV | 0.007-70 | 0.32×10^-5^ | Serum and drinking bottle |
| MIP/Au@PTH (Chai and Kan, 2019) | CV/DPV | 0.08-100 | 38 | Tap and drinking water, river water |
| MIP/PPy/GQDs (Tan et al., 2016) | CV | 0.1–50 | 40 | Tap and sea water |
| MIP/PPy/LSG (Beduk et al., 2020) | DPV | 0.05–5 | 8 | Tap water, PC, PET water bottle |
| MIP/ BOMC/Au@CNT (Hu et al., 2018) | DPV | 0.01-10 | 5 | Milk |
| mMIP@mGCE (Lu et al., 2021) | CV/DPV |  | 133 | Tap water, municipal sewage, tea drink, milk, cabbage and soil |
| MIP/Chitosan (Galai et al., 2020) | SWV | 1000-10^-15^ | 6.7×10^-11^ | Plastic bottles |
| MIP/ERGO/GCE (Karthika et al., 2021) | CV/EIS | 0.75-0.0005 | 0.2 | Tap water, skimmed bovine milk, baby bottles |
| MIP/CdTe @ QDs-MWCNTs (Zhang et al., 2021b) | CV/DPV/EIS | 0.00005-0.05 | 0.005 | Water samples |
| MIP/AuNPs/MCA/rGO/CILE (Jalilian et al., 2021) | CV/EIS | 18-0.004 | 1.1 | Plastic containers |
| MIP/CDs (Zhang et al., 2021a) | Fluorescence spectra | 0.02-2mgL^-1^ | 0.016 mgL^-1^ | Canned fruit, meat and drink samples |
| Laccase–thionine–CB/GCE (Portaccio et al., 2013) | CV | 0.5–5.0 | 200 | Tomato juice |
| AuNDs/CTAB/GCE (Lien et al., 2020) | DPV | 0.025-10 | 22 | Water bottle |
| NP-PtSi/GR/GCE (Zhang et al., 2020b) | DPV | 0.3-85 | 0.11 | Milk |
| Ce-Ni-MOF (Huang et al., 2020) | CV and DPV | 0.1-100 | 7.8 | Bottled water |
| BCNPs/Tyr/Nafion/GCE (Liu et al., 2019) | CV | 0.02-10 | 3.18 | Ground water |
| AgNPs/GPUE (Baccarin et al., 2020) | DPV | 2.5–15 | 240 | Tap and river water |
| AuNP/SGrNF/GCE (Niu et al., 2013) | LSV | 0.08–250 | 35 | Baby bottles |
| NH_2_-MIL-125/rGO/GCE (Ling et al., 2018) | CV | 2–200 | 796 | Drinking bottles, packaging and paper cups |
| AuNP/mCNT@ZIF-8 (Li et al., 2021) | CV | 1–100 | 690 | Pond, tap and river water |
| PANINR/MWCNT/PGE (Poorahong et al., 2012) | CV | 1.0–400 | 10 | Baby bottles |
| CAS/CB/GCE (Vieira Jodar et al., 2019) | DPV | 0.49–24 | 250 | Tap water and milk |
| GNPs/ MWCNTs (Li et al., 2016) | LSV | 0.87–87 | 130 | Plastic samples |
| NiFe_2_O_4_/rGO/SPE (Bas et al., 2021) | DPV | 0.05–25 | 10 | Water bottle |
| NiNP/NCN/CS/GCE (Wang et al., 2020) | DPV | 0.1–2.5 | 45 | Milk |
| AuNPs/LSG-MIP | **DPV** | **0.01-10** | **3.97** | **Bottled water, tap water, milk, baby formula, plastic samples.** |
|  |  |  |  |  |
|  |  |  |  |  |

Abbreviations: mGCE- Magnetic glassy carbon electrode; NiNP- nickel nanoparticles; NCN nitrogen-doped carbon nanosheet; CS- chitosan; FTO- Fluoride doped tin oxide; GCE- glassy carbon electrode; NiFe_2_O_4_- nickel ferrite; rGO- reduced graphene oxide; CAS- casein; CB- Carbon Black; CBNP- Carbon black nanoparticle; GNPs- graphene nanoplatelets; mCNT- magnetic carbon nanotubes; PANINR-polyaniline nanorod; PTH- polythionine; PPy- Polypyrrole; PGE -Pencil graphite electrode; AuPdNP- Gold Paladium nanoparticles; g-C3N4r - graphitic carbon nitride; BOMC- boron-doped ordered mesoporous carbon; mMIP- Magnetic molecularly imporinting polyer; GrN-graphene nanosheets; NH2-MIL-125-amine-functionalized metal–organic framework; ERGO- electrochemically reduced graphene oxide; MCA- Mercaptamine; CILE- Carbon ionic liquid electrode; CD- Carbon dots; CTAB- cetyltrimethylammonium bromide; SPE-screen printed electrode; BCNP- Biochar nanoparticle, MWCNT-multiwalled carbon nanotubes; GPUE- Graphite:polyurethane; GQDs- Graphene quantum dots SWCNT- single-walled carbon nanotubes; MIPPy- molecularly imprinted polypyrrole; SGrNF - Stacked graphene nanofibers.

**References**

Anirudhan, T.S., Athira, V.S., and Chithra Sekhar, V. (2018). Electrochemical sensing and nano molar level detection of Bisphenol-A with molecularly imprinted polymer tailored on multiwalled carbon nanotubes. *Polymer* 146**,** 312-320.

Baccarin, M., Ciciliati, M.A., Oliveira Jr, O.N., Cavalheiro, E.T., Raymundo-Pereira, P.a.J.M.S., and C, E. (2020). Pen sensor made with silver nanoparticles decorating graphite-polyurethane electrodes to detect bisphenol-A in tap and river water samples. 114**,** 110989.

Bas, S.Z., Yuncu, N., Atacan, K., and Ozmen, M. (2021). A comparison study of MFe2O4 (M: Ni, Cu, Zn)-reduced graphene oxide nanocomposite for electrochemical detection of bisphenol A. *Electrochimica Acta* 386**,** 138519.

Beduk, T., Ait Lahcen, A., Tashkandi, N., and Salama, K.N. (2020). One-step electrosynthesized molecularly imprinted polymer on laser scribed graphene bisphenol a sensor. *Sensors and Actuators B: Chemical* 314**,** 128026.

Ben Messaoud, N., Lahcen, A.A., Dridi, C., and Amine, A. (2018). Ultrasound assisted magnetic imprinted polymer combined sensor based on carbon black and gold nanoparticles for selective and sensitive electrochemical detection of Bisphenol A. *Sensors and Actuators B-Chemical* 276**,** 304-312.

Chai, R., and Kan, X. (2019). Au-polythionine nanocomposites: a novel mediator for bisphenol A dual-signal assay based on imprinted electrochemical sensor. *Analytical and Bioanalytical Chemistry* 411**,** 3839-3847.

Galai, H.C., Namour, P., Bonhomme, A., Bessueille, F., Hentati, S.B., and Jaffrezic-Renault, N.J.J.O.T.E.S. (2020). Elaboration of an imprinted polymer film based on chitosan electrodeposition for the voltammetric detection of BPA. 167**,** 027507.

Hu, X., Feng, Y., Wang, H., Zhao, F., and Zeng, B. (2018). A novel bisphenol A electrochemical sensor based on a molecularly imprinted polymer/carbon nanotubes-Au nanoparticles/boron-doped ordered mesoporous carbon composite. *Analytical Methods* 10**,** 4543-4548.

Huang, X., Huang, D., Chen, J., Ye, R., Lin, Q., and Chen, S. (2020). Fabrication of novel electrochemical sensor based on bimetallic Ce-Ni-MOF for sensitive detection of bisphenol A. *Analytical and Bioanalytical Chemistry* 412**,** 849-860.

Jalilian, R., Ezzatzadeh, E., and Taheri, A. (2021). A novel self-assembled gold nanoparticles-molecularly imprinted modified carbon ionic liquid electrode with high sensitivity and selectivity for the rapid determination of bisphenol A leached from plastic containers. *Journal of Environmental Chemical Engineering* 9**,** 105513.

Jebril, S., Cubillana-Aguilera, L., Palacios-Santander, J.M., and Dridi, C. (2021). A novel electrochemical sensor modified with green gold sononanoparticles and carbon black nanocomposite for bisphenol A detection. *Materials Science and Engineering: B* 264**,** 114951.

Karthika, P., Shanmuganathan, S., Viswanathan, S., and Delerue-Matos, C. (2021). Molecularly imprinted polymer-based electrochemical sensor for the determination of endocrine disruptor bisphenol-A in bovine milk. *Food Chemistry* 363**,** 130287.

Li, H., Wang, W., Lv, Q., Xi, G., Bai, H., and Zhang, Q.J.E.C. (2016). Disposable paper-based electrochemical sensor based on stacked gold nanoparticles supported carbon nanotubes for the determination of bisphenol A. 68**,** 104-107.

Li, H., Zhu, F., Xiang, J., Wang, F., Liu, Q., and Chen, X. (2021). In situ growth of ZIF-8 on gold nanoparticles/magnetic carbon nanotubes for the electrochemical detection of bisphenol A. *Analytical Methods* 13**,** 2338-2344.

Lien, N.T., Quoc Hung, L., Hoang, N.T., Thu, V.T., Ngoc Nga, D.T., Hai Yen, P.T., Phong, P.H., and Thu Ha, V.T. (2020). An Electrochemical Sensor Based on Gold Nanodendrite/Surfactant Modified Electrode for Bisphenol A Detection. *Journal of Analytical Methods in Chemistry* 2020**,** 6693595.

Ling, L.J., Xu, J.P., Deng, Y.H., Peng, Q., Chen, J.H., San He, Y., and Nie, Y.J. (2018). One-pot hydrothermal synthesis of amine-functionalized metal–organic framework/ reduced graphene oxide composites for the electrochemical detection of bisphenol A. *Analytical Methods* 10**,** 2722-2730.

Liu, Y., Yao, L., He, L., Liu, N., and Piao, Y. (2019). Electrochemical Enzyme Biosensor Bearing Biochar Nanoparticle as Signal Enhancer for Bisphenol A Detection in Water. *Sensors (Basel, Switzerland)* 19**,** 1619.

Lu, Y.C., Xiao, W.W., Wang, J.Y., and Xiong, X.H. (2021). Rapid isolation and determination of bisphenol A in complicated matrices by magnetic molecularly imprinted electrochemical sensing. *Analytical and Bioanalytical Chemistry* 413**,** 389-401.

Niu, X.L., Yang, W., Wang, G.Y., Ren, J., Guo, H., and Gao, J.Z. (2013). A novel electrochemical sensor of bisphenol A based on stacked graphene nanofibers/gold nanoparticles composite modified glassy carbon electrode. *Electrochimica Acta* 98**,** 167-175.

Poorahong, S., Thammakhet, C., Thavarungkul, P., Limbut, W., Numnuam, A., and Kanatharana, P. (2012). Amperometric sensor for detection of bisphenol A using a pencil graphite electrode modified with polyaniline nanorods and multiwalled carbon nanotubes. *Microchimica Acta* 176**,** 91-99.

Portaccio, M., Di Tuoro, D., Arduini, F., Moscone, D., Cammarota, M., Mita, D.G., and Lepore, M. (2013). Laccase biosensor based on screen-printed electrode modified with thionine–carbon black nanocomposite, for Bisphenol A detection. *Electrochimica Acta* 109**,** 340-347.

Tan, F., Cong, L.C., Li, X.N., Zhao, Q., Zhao, H.X., Quan, X., and Chen, J.W. (2016). An electrochemical sensor based on molecularly imprinted polypyrrole/graphene quantum dots composite for detection of bisphenol A in water samples. *Sensors and Actuators B-Chemical* 233**,** 599-606.

Vieira Jodar, L., Orzari, L.O., Storti Ortolani, T., Assumpção, M.H.M.T., Vicentini, F.C., and Janegitz, B.C. (2019). Electrochemical Sensor Based on Casein and Carbon Black for Bisphenol A Detection. *Electroanalysis* 31**,** 2162-2170.

Wang, Y., Yin, C., Zhuang, Q.J.J.O.A., and Compounds (2020). An electrochemical sensor modified with nickel nanoparticle/nitrogen-doped carbon nanosheet nanocomposite for bisphenol A detection. 827**,** 154335.

Wang, Z., Yan, R., Liao, S., Miao, Y., Zhang, B., Wang, F., and Yang, H. (2018). In situ reduced silver nanoparticles embedded molecularly imprinted reusable sensor for selective and sensitive SERS detection of Bisphenol A. *Applied Surface Science* 457**,** 323-331.

Yan, K., Yang, Y., Zhang, J.J.S., and Chemical, A.B. (2018). A self-powered sensor based on molecularly imprinted polymer-coupled graphitic carbon nitride photoanode for selective detection of bisphenol A. 259**,** 394-401.

Zhang, J., Wang, H., Xu, L., and Xu, Z. (2021a). A semi-covalent molecularly imprinted fluorescent sensor for highly specific recognition and optosensing of bisphenol A. *Analytical Methods* 13**,** 133-140.

Zhang, R.-R., Zhan, J., Xu, J.-J., Chai, J.-Y., Zhang, Z.-M., Sun, A.-L., Chen, J., and Shi, X.-Z. (2020a). Application of a novel electrochemiluminescence sensor based on magnetic glassy carbon electrode modified with molecularly imprinted polymers for sensitive monitoring of bisphenol A in seawater and fish samples. *Sensors and Actuators B: Chemical* 317**,** 128237.

Zhang, S., Shi, Y., Wang, J., Xiao, L., Yang, X., Cui, R., and Han, Z. (2020b). Nanocomposites consisting of nanoporous platinum-silicon and graphene for electrochemical determination of bisphenol A. *Microchimica Acta* 187**,** 241.

Zhang, Y., Huang, W., Yin, X., Sarpong, K.A., Zhang, L., Li, Y., Zhao, S., Zhou, H., Yang, W., and Xu, W. (2020c). Computer-aided design and synthesis of molecular imprinting polymers based on doubly oriented functional multiwalled carbon nanotubes for electrochemically sensing bisphenol A. *Reactive and Functional Polymers* 157**,** 104767.

Zhang, Y., Zhang, W., Zhang, L., Song, G., Wang, N., Xu, W., and Huang, W. (2021b). A molecularly imprinted electrochemical BPA sensor based on multi-walled carbon nanotubes modified by CdTe quantum dots for the detection of bisphenol A. *Microchemical Journal* 170**,** 106737.

Zheng, W., Xiong, Z., Li, H., Yu, S., Li, G., Niu, L., and Liu, W. (2018). Electrodeposited Pt@Molecularly imprinted polymer core-shell nanostructure: Enhanced sensing platform for sensitive and selective detection of bisphenol A. *Sensors and Actuators B: Chemical* 272**,** 655-661.
